# Abstracts and Gleanings

**Published:** 1888-10

**Authors:** 


### ABSTRACTS AND GLEANINGS.

### SUMMER DIARRHŒAS OF INFANCY AND CHILDHOOD.

By Louis Starr, M. D., Philadelphia.

The diarrhœal affections of hot weather may be grouped under two heads, namely: 1. Ordinary summer diarrhœa, or entero-colitis 2. Cholera infantum. The former is the more common and the more manageable, and so far from being a mild type of the latter, is a distinct disease, requiring its own methods of treatment.

1. Summer diarrhœa, or entero-colitis. Therapeutic measures often fail in relieving this condition when uncombined with rigid enforcement of the general rules of health. The main hygienic features to receive attention are the following: Fresh air must be secured in the cool of the morning and evening, or better still, by a morning or evening trip on the water. The heat of the day must be spent in as cool a room as can be had. Coddling is to be discouraged, as many a stout mother has hastened her infant's death by too fond and constant nursing in the arms. The clothing must be as thin as possible, provided that woolen be always worn next to the skin. Twice or three times a day in very hot weather, the whole surface of the body must be sponged with water at a temperature of 80° Fahr., and dried with gentle rubbing. The addition of rock salt renders these baths more bracing. Full warm baths must supplant the cold spongings if there be much prostration.

The diet is to be most carefully regulated as to quality, quantity, and intervals of administration. Sound cow's milk must form the basis of the food in bottle-fed babies, and peptogenic powder is a very useful addition to it.

Medicinal treatment varies with the case. Should the patient be seen early in the attack, it is initiated by a laxative. A teaspoonful of castor oil with 10 drops of paregoric, or the same quantity of spiced syrup of rhubarb, is sufficient for an infant of one year. Afterward while the stools are yellow, homogeneous, and not very frequent, alkalies and astringents are employed:

℞. Sodii bicarb..............................gr. xxxvi,

Syr. rhei aromat..........................ʒ iv,

Mist. cretae, q. s. ad.......................ʒ xxiv.

M. S. One teaspoonful every two hours for a child of one year.

When the stools are frequent, green and acid in reaction, the the following may be employed:

℞. Syr. rhei aromat..........................ʒ iv,

Bismuth subcarb.............................ʒ ij,

Syrupi acacia...............................ʒ iv,

Mistura creta, q. s. ad.......................ʒ xxiv.

M. Sig.—One dram every two hours. At the same time the abdomen is to be reddened two or three times a day, with a weak mustard draught—one part of mustard to five of flour.

If the evacuations be liquid and contain whitish or greenish flakes, and the above treatment fail after a fair trial, good results often follow a short mercurial course, thus:

℞. Pulv. ipecac. comp. . . . . . . . . . . . . . . . . . . . . . . .gr. ij,
Hydrarg. chlor. mit. . . . . . . . . . . . . . . . . . . . . . . .gr. ss,
Creta preparat. . . . . . . . . . . . . . . . . . . . . . . . . . .gr. xxxvi.

M. ut ft. chart, No. 12. S.—One powder every two hours for twenty-four or forty-eight hours, or until the stools become yellow or homogeneous.

Should the stools be frequent and serous, more powerful astringents are used, as aromatic sulphuric acid, silver nitrate, or zinc oxide. When the stomach is very irritable, rectal injections are resorted to, the drugs used being tincture of opium. nitrate silver and ipecacuanha is chosen where there is much tenesmus with the discharge of blood and mucus. It may be administered as follows:

℞. Ext. ipecac fl. . . . . . . . . . . . . . . . . . . . . . . . . . . . . . .*m.* xii,
Tr. opii deod. . . . . . . . . . . . . . . . . . . . . . . . . . . . . . . .*m.* viij,
Mucilag. acacia, q. s. ad. . . . . . . . . . . . . . . . . . . . . . .ʒ 8.

M. S. Inject 1 tablespoonful every four hours.

Stimulants—wine of pepsin, brandy or whisky—are given in all infantile cases where there is prostration.

In cases of recovery, the diet and hygiene must be carefully watched until all danger of a relapse has passed, the astringents are gradually dropped, and digestants and tonics ordered.

The antiseptic treatment recommended by L. Emmet Holt, I have lately tried with good results. It embraces the careful attention to regimen already alluded to, preliminary evacuation of the bowels with castor oil, and the administration of naphthalin or of sodium salicylate. Naphthlin is usually ordered as in the following prescription:

℞. Naphthalin. . . . . . . . . . . . . . . . . . . . . . . . . . . . .

Ground coffee. . . . . . . . . . . . . . . . . . . . . . . . . . .aa gr. vi,

Sugar of milk. . . . . . . . . . . . . . . . . . . . . . . . . . . .gr. xxiv.

M. ut ft. chart No 12. S.—One powder every two hours.

In conclusion it may be well to draw attention to the fact that the key note of successful treatment seems to be the maintenance of constant circulation in the contents of the intestinal tract. The object of this is to sweep irritating fæcal matter or secretions away from the intestinal mucous membrane and give the latter an opportunity to recover from the catarrahl inflammation affecting it Castor oil and calomel are the best drugs to accomplish this, and small, frequently repeated doses are to be preferred to single large ones, active purgation being undesirable. With bowels so swept, a bland, unfermenting diet, and attention to the health rules already mentioned, every aid is furnished to secure the successful action of such remedies as bismuth subcarbonate, naphthalin and sodium salicylate.

On the other hand, should opium be used to lock the bowels, one great factor in the causation of the disease is fortified in its position, and an increase in the degree of inflammation almost invariably results. The opium used in the foregoing prescriptions is only intended to prevent griping or to secure retention in the case of the injection; not for the purpose of checking peristaltic action of the intestine.

In some cases, particularly where there is irritability of the stomach, milk in no matter what form or how prepared, seems to keep up the disease. Under these circumstances my plan is to order one or two teaspoonsful of raw beef juice every two hours, according to age. This diet may be continued for several days, until vomiting stops and the movements improve in character, when a milk diet may be resumed.

One must not forget that a change of climate is a most efficient method of treatment, especially when the seaside is the objective point.

2. Cholera infantum: The large and frequent watery evacuations characteristic of this disease are such a drain upon the system that it is of the first consequence to replace the waste by food and drink, and at the same time check it by appropriate treatment. The irritability of the stomach is a formidable barrier to alimentation, nevertheless every effort must be made to give food in small quantities and at short intervals. Should the infant be at the breast, it may be allowed to nurse for a few minutes every half hour or hour. If hand-fed it may be given the foods suitable in entero-colitis, or in chronic vomiting, in such quantities as can be retained and at intervals corresponding in frequency to the smallness of the amount. Bits of ice and water should be allowed freely, even though they be rejected as soon as swallowed.

To check the diarrhœa opium and astringents are necessary. A very serviceable formula is the following:

℞. Liquor morphine sulphat .................... ʒ j,

Acid sulphuric aromat ........................ *m* xxiv,

Elix. curacoæ ................................ ʒ iv,

Aquæ, q. s. ad ............................... ʒ xxiv.

M. Sig.—One teaspoonful every two hours for a child two months old.

With this, 2 drops of laudanum, suspended in 2 teaspoonsful of starch water should be given by the rectum every three hours. Two or three times daily a mustard plaster, one part of mustard and five of flour, must be applied over the whole surface of the abdomen long enough to redden the skin, and the whole body should be sponged several times a day with water at a temperature of 95° F.

The clothing, diapers and person must be kept perfectly clean, the sick room must be as large and airy as can be commanded, and the infant must lie upon a bed and not be constantly nursed on the lap. If it be possible, the patient should be sent early to the seashore or country, as this affords by far the best chance of recovery. Failing in this, morning and evening airings in a coach or daily steamboat excursions must be resorted to.

Stimulants are needed from the first, to ward off prostration—from 5 to 10 drops of whisky in a teaspoonful of limewater may be given every two or three hours, at the age of six months.

When collapse sets in, the quantity of alcohol must be increased, and, if the stomach can bear it, a combination of stimulants is useful, as

| | | |
|---|---|---|
| ℞. | Spt. frumenti | ʒ iv, |
| | Ammon. carbonatis | gr. xxiv, |
| | Syr. acaciæ | ʒ viij, |
| | Aq. menthæ pip. q. s. ad | ʒ xxiv. |

M. Sig. One teaspoonful p. r. n.

The temperature must be maintained by hot flannel wraps and hot water bottles, and the child be kept in a horizontal position and disturbed as little as may be.

In this stage astringents are still indicated, but opium must be used with great caution, or even discontinued entirely, when there are cerebral symptoms and semi-coma.

In the fortunate instances, in which this plan is successful, it is necessary to treat the succeeding diarrhœa, and to build up the general health by good food, tonics and fresh air.—*Med. Standard.*

---

**Antiseptic Action of Chloroform Water.**—This useful property of chloroform is well illustrated in the *Deutsche med. Wochenschrift*, Heft, 19. Prof. Salkowski calls attention to the powerful antiseptic properties of chloroform water, which he extols in no mistakable terms. Having used it for years to prevent the decomposition of urine, special experiments have shown him that, if the chloroform be kept from evaporating, it stops all fermentative processes conditioned upon the vital activity of micro-organisms; thus, milk keeps for months its original neutral and alkaline reaction. Solutions of cane and grape sugars, mixed with yeast, do not ferment. Albuminous solutions, meat-juice keep perfectly sterile; while ordinary solutions, used for purpose of control, showed bacteria in two days. It is, too, a disinfectant as well as an antiseptic. Very stinking meat juice, shaken with chloroform water, was sterile after one hour's standing. Anthrax bacilli were innocuous after twenty-four hours' contact, and a culture of cholera coma bacillus was made inert in a minute.

From this experimental evidence Salkowski draws the following practical hints and urges experimental trial of chloroform water:

1. Chloiorm water in the laboratory is a decidedly superior agent to add to all ferment solutions, albuminous fluids, extracts, etc., it is far ahead of any other antiseptic. Here its volatility is of great advantage, permitting its removal by heat or air current when necessary. To preserve urine unchanged it is of great value. In urine already alkaline chloroform will not act on the *soluble* ferment present.

2. For the preservation of smaller anatomical preparations; the only drawback is its taking up the blood-coloring matter, a difficulty which further experience may obviate.

3. In therapeutics as a solvent for alkaloids in solutions; subcutaneous injection, for the irritating effects of the chloroform are slight. With so few antiseptics of disinfection of the alimentary canal among our resources, this adaptation of it should be borne in mind.

Animals bear very large quantities (a dog got for four days in succession 7 ounces in his food, without any effect). In cholera it should certainly be tried. Exceptionally in emergencies it might be used as an external antiseptic, though inferior to others. Its easy preparation (*m*lxxv X 75!), shaken in a quart of water) is a recommendation.

[This proportion of chloroform is rather large, as about 1 part of chloroform to 200 of water is as much as will dissolve.—ED.]

A similar laudatory estimate of the value of chloroform in pharmacy for the preservation of extracts, making solutions of drugs, etc., may be found in the *American Journal of Pharmacy*, May, 1888, p. 248.

Unna (*Monatsh. f. prakt. Dermat.*, 1888, Heft 9), on the recommendation of Hager, has used aqua chloroformi as a vehicle for subcutaneous solutions, while the chloroform, though it causes a slight burning sensation in the morphia solution, is of advantage through its anæsthetic properties in such solutions as that of ergotin and in Fowler's. For these he recommends it especially.—*Amer. med. Jour. Sciences.*

**Etiology and Treatment of Yellow Fever.**—(Gibier, in *LaFrance Medicale.*) In a previous article the result of some researches made upon yellow fever at Havana were presented to the Academy, and he now gives a resume of all the observations made since.

1. In a very large majority of the cases in which examinations have been made of the blood, bile, urine, the pericardiac serosity and the viscera (with the exception of the digestive tube), there were found no micro organisms. So that it may be asked if it is not likely that in the rare cases in which microbes are met it is not possible that they were accidentally introduced in the cultures, particularly as the species are *variable*. However, it can be admitted as possible that they may have been introduced into the circulation accidentally by means of intestinal lesions.

2. The intestines of subjects attacked by yellow fever contain a black or dark matter, more or less abundant and toxic, as shown by experiment.

3. From this black matter taken from the intestine he has isolated a bacillus, which seems to play an important *role* in the coloration of this substance, if not in the pathology of yellow fever. This microbe blackens the body in which it developes. It is a bacillus sometimes straight and short, sometimes a little longer and curved. It liquefies gelatine. Inoculation of the intestines of animals (dogs and foxes) with a small quantity of the culture of this liquid, produces grave effects, and even death, with the formation in the intestine of a matter like that found in men dying from yellow fever. The other characteristics of yellow fever are as follows: The cultures exhale an odor *sui generis*, and like that of black vomit. A temperature of 60° C. destroys it in ten minutes; a cold of 10° below zero continued for an hour does not kill it.

Drying in the open air in the shade kills it in twenty-four hours. It cultivates well in sea water and lives at least six months, according to my observation, in contact with ordinary microbes.

A temperature above 20° is necessary to its development.

It does not produce spores. The long, undulating form which it takes in old cultures would seem to class it with the spirilli.

If this bacillus is indeed that which causes the phenomena of yellow fever, the preceding characteristics will explain the fact that this disease is only observed endemically in a certain number of seaports of warm countries, where the soil contains the germ of a malady comparatively unknown at a short distance inland.

4. The constant presence in the intestines of matter more or less abundantly *toxic*, the early occurrence of gastro-intestinal troubles (vomiting, epigastric pain, etc.), which usually persist during the entire course of the sickness; the brusque onset of these symptoms, the absence of microbes in the blood and in the viscera, other than the intestines, are other characteristics which militate in favor of the intestinal theory of yellow fever, and if this theory is in accord with the facts, the treatment which I have indicated in a conference with the physicians at Havana (repeated purgatives and intestinal disinfectants), should be successful in the disease which it is intended to combat. Inversely, if a grave case in the civil hospital of Havana, to whom this treatment was given with success, does not remain unique, the intestinal theory of yellow fever may be considered established. *Naturæm morboram remedia demonstrant.—Medical Times.*

**The Boracic-Acid-Leucorrhœa-Treatment.**—Harriet C. B. Alexander, M. D., Chicago, Physician to Cloverly Home for Nervous Invalids, says: Dr. N. F. Schwartz, of St. Louis, (*Med. Standard*) has claimed remarkable results in the treatment of leucorrhœa from the use of boracic acid. The method advocated by Dr. Schwartz is that followed in otitis media purulenta, and it was the success in its use in the latter disease which led him to try boracic acid in leucorrhœa. He first irrigates the vagina with water as hot as can be borne, then a speculum is introduced and the vaginal walls are carefully dried with absorbent cotton pledgets. Sufficient boracic acid is poured through a cylindrical glass speculum to completely distend the vaginal vault and surround the cervico-vaginal portion.

Dr. H. N. Moyer, who has tried Dr. Schwartz's procedure with success, greatly prefers the larger crystals, as they dissolve slowly, and the effect is more prolonged. The crystals are held firmly in in place by small absorbent cotton tampons supported by a large aseptic wool tampon.

The treatment was tried by myself in three cases, and in all with good results. The first case was that of a twenty-two year old neurasthenic woman whose general health had become much impaired. She suffered, at the time I saw her, from various imperative conceptions, prominent among which were those of mysophobia. She was the victim of a bad heredity and exhaustion from physical disease. She was treated with cold baths and general tonics, since for moral reasons it was deemed advisable to desist from any treatment of the reproductive apparatus until her geneneral health and mental condition had improved. The patient's will-power was strengthened and her general health had greatly improved when she complained of a profuse leuchorrhœa, which on examination was found to be due to endometritis. I thrice packed her vagina with boracic acid, in the manner already indicated by Dr. Schwartz. After the second packing a hardening of the cervix was noticeable, which was still more marked after the third. She recovered without any apparent untoward results from her leuchorrhœ.

The second case was that of a married woman who suffered from leucorrhœa dependent on endometritis. The discharge became profuse after menstruation or unusual exertion. After the third packing I noticed that the cervix, which had been denuded of epithleum, had a line of healing about a quarter of an inch wide and was decidedly hardened, but the leucorrhœa had entirely ceased.

How long this hardening persists after the boracic acid treatment I am unable to state, as since recovery from leucorrhæa I have not had the opportunity of examining either case. All three patients made a good recovery from leucorrhœa and were pleased with the treatment. Since this hardening of the cervix has not been discussed in connection with the boracic acid treatment, I have thought it of sufficient interest to merit mention.—*Epitome.*

**Treatment of Carcinoma Uteri.**—(Schauta, *Centrlabl. f. Gyn.*, February 4, 1888).—The relative value of partial and complete extirpation of the uterus is discussed in this paper, and a conclusion reached in favor of the latter. The author analyzes the statistics of Hofmeier, who advocates, with Schroder, the partial operation, and alludes to the statement that the total extirpation is advised by them only in cases in which the disease has extended beyond the os internum. In such cases, however, the disease is well advanced, and it is easy to understand that the comparative results must be unfavorable to the radical operation. A more satisfactory opinion can be reached by comparison of the statistics of operators who do both the partial and complete operations with those of the ones who do only the complete. Thus, by comparison of the statistics of Hofmeier with those of Martin, it is seen that in the former there has been 45 per cent. of permanent recoveries, and in the latter 70 per cent. As to the charge that there is much greater immediate danger from the complete operation, the statistics of Martin and Fritsch for the past two years furnish a sufficient denial. The assertion that removal of the entire uterus removes the possibility of conception and child-bearing is counterbalanced by the fact that those who have become pregnant after the partial operation have always aborted. As an illustration that it is impossible to distinguish clinically the limits of carcinoma of the cervix, the author cites three cases which came under his observation. In a woman who had reached the sixth month of pregnancy, carcinoma of the anterior lip of the cervix was diagnosticated, and the lip was excised. Normal labor occurred at term. Three years and a quarter later, carcinoma appeared in the posterior lip. The uterus was extirpated, and there has been no recurrence. In a woman forty-seven years of age, a tumor of the cervix as large as an apple, and apparently well defined, was observed. After total extirpation, it was found that the disease had gone beyond the os internum. In a third case, in which extirpation was performed on account of a very small superficial carcinomatous growth of the cervix, a small carcinomatous nudle was also found at the fundus. Clinical facts teach us that the greatest care and attention should be paid to cases in which there are catarrh and erosion of the uterus. If a nodule is at any time discovered, it should be at once excised, and examined microscopically for carcinomatous elements. By this method the disease may be discovered, in many cases, in its early and curable stage.—*N. Y. Medical Journal.*

**Saccharin.**—According to the latest analysis, saccharin is a white powder, showing under the microscope a crystalline form and soluble in eighty per cent. alcohol or in hot water. It does not reduce Fehling's solution. It suffers no change in the system, and its elimination by the urine commences in half an hour (*Deut. med. Zeit.*, February 23, 1888, p. 197). The substance was first obtained in 1879 from coal tar, and, on account of its intense sweetness (two hundred and eighty times that of sugar—one to two grains sweeten a cup of coffee), has come to be liberally used in manufactures—*e. g.*, beer.

In medicine its chief uses have been in diabetes, in place of cane sugar and as a corrigent. It has been looked upon as an indifferent substance in its effect on the system. Thus, Prof. Mosso gave it in large quantities to animals without damaging results, and 75 grains in men produced no bad effects. Salkowski (Virchow's *Archiv*, cv. p. 46) confirmed its innocuousness. Stadelman, employing it on eleven patients, in doses of 50 to 75 grains, found it harmless in nine, while in two others it caused severe gastric disturbance.

Saccharin has antiseptic properties, and Mosso noticed the fact that the urine of animals taking it kept longer. Little (*Dublin Journal of Medical Science*, June, 1888, p. 493) has reported his use of it in ammoniacal urine dependent on paralysis of the bladder, on stricture or on prostatic disease. The results were highly satisfactory in a series of half a dozen cases. In one case of multiple calculi,, in an old lady in which operation was refused, the urine, which had been intolerably putrid for three months, became non-ammoniacal and sweet in three or four days, and continued so. Little has not found it upsetting to the stomach; in fact, he emphasizes this point of advantage over other drugs given to improve the urine.

Eichhorst (quoted in *Munch. med. Wochsft.*, July 10, p. 478) in the treatment of diabetes, finds saccharin of much worth, but warns against its use in too large quantities, as it easily produces an unpleasant after-taste, nausea and disgust for the medicine.

**Treatment of Dysentery**—As an article of diet, besides the list of farinaceous foods and meat juices, I wish to emphasize one substance above all others; I refer to buttermilk. With an experience through a number of epidemics, and in many sporadic and malarial cases since 1857, when I began the use of this Southern drink, I am prepared to conclude that in addition to the qualities of nutrition it possesses curative properties. I have never seen harm arise from its use when there did not exist an idiosyncrasy in health. My attention was directed to the article by a paper written by Dr. Stephens, of Chester, Pa., which was published in the "*American Journal of the Medical Sciences*" during the year above mentioned. Let me reiterate and further emphasize the use of buttermilk in dysentery, in whatever form the disease may be encountered. "Stephenize" the patient from beginning to end of case. It is grateful to most persons, and a valuable adjuvant to whatever therapy may be adopted.

Dr. Edwards said: "Doctor, I never lost a case of dysentery, and I always treated and cured such cases by saturating equal parts of apple vinegar and mint water with common salt, giving a dose every two or three hours."

Upon one occasion I was treating a very severe case of this disease. I had "wrung the changes," did the best my judgment would suggest, but the patient got no better. I wrote the following and sent the husband to the nearest drug store for the medicine. The patient made an uninterrupted and rapid recovery.

℞. Acidi acetici..................................
Aquæ menthæ piperitæ.......................aafl ℥ j,
Sodii chloridi....................ad. saturandum.

M. et S.—Dessertspoonful every two or three hours.

I ordered buttermilk—this was my hobby; the vinegar and salt was Dr. Edwards'. Which cured the patient?—*E. J. Bell*, *Texas Courier-Record of Medicine.*

**Carbolic Acid in Treatment of Typhoid Fever.**—In the *Lancet* Dr. F. Sidney Gramshaw gives his experience with the use of carbolic acid in one hundred and sixteen cases of enteric fever.

The diet is restricted to milk toast and water, barley water, and calf's-foot jelly; new milk is always insisted upon as the main support, from a quart to three pints being given to an adult in the twenty-four hours. The carbolic acid is ordered in a mixture, of which this is the prescription: Take of carbolic acid (Calvert's extra pure for internal administration), 12 minims; tincture of iodine (B.P.), 16 minims; tincture of orange-peel, 1½ drachm; simple syrup, 3 drachms; water to 8 ounces; the dose to be an ounce every four hours for the first fortnight, or until the urgent symptoms yield, when same dose is administered three times a day. The good effect is manifested almost immediately. In two days the pulse slows and gains in strength, the temperature falls, the tongue becomes moist, all diarrhœa ceases, and the general condition of the patient is so much improved that, as a rule, in a week all anxiety is at an end, and the case progresses quietly towards recovery. It sometimes happens that a case is cut short by this treatment as suddenly as is a case of acute rheumatism by the exhibition of salicylate of sodium; but more generally the fever runs its course of thirty days before all danger of relapse is past, and he found it better to continue the medicine until the thermometer shows no rise of temperature for three or four clear days.

Beef tea is carefully avoided so long as the temperature is abnormal, because it so frequently gives rise to a troublesome diarrhœa. A day or two after commencing the carbolic acid mixture patients always complain that everything they take tastes of the medicine. This is unavoidable, and need give no anxiety unless vomiting is excited, when it is a good plan to reduce the dose of carbolic acid and to add a small quantity of dilute nitro hydrochloric acid.—*Practice.*

**Antipyrin in the Treatment of Seminal Emissions.**—The older remedies for this affection, camphor and lupulin, have very properly been abandoned. Kurschmann says that the sedative action of lupulin on the genital organs is far from demonstrated, and the employment of camphor is not more reliable, although Zeissl, Purjez and others consider it the best remedy in this affection. Nux vomica, arsenic and atropine have also been recommended, while Diday prefers the bromides of potassium and sodium to all other remedies. He recommends from 30 to 80 grains of the bromide of potassium to be taken on retiring. But these large doses of bromide will produce acne, and are also liable to induce mental enfeeblement. In order to avoid the dangers of bromides, Thor, of Bucharest, has been experimenting with antipyrin in the treatment of these affections. He advises the patient to take from 7 to 15 grains of the drug on retiring. In seventeen cases he has completely cured the complaint, without any unpleasant consequences. According to Beart, antipyrin is useful in neurasthenia of the sexual organs, but in these cases from 1 to 2 grains a day should be given.—*Revista de Ciencias de Medicas.*

**One-Child Sterility.**—A considerable proportion of women never bear more than one child, and this fact is easily explained, for pregnancy may be complicated or followed by disorders which damage the genital apparatus. Dr. Kleinwachter (*Zeitschrift fur Heilkunde*) observed 90 cases where women had been but once pregnant, and on that occasion at a more or less per od, out of 1,081 patients in his gynecological practice. In 21 of the 90 cases the pregnancy had ended prematurely. He classed the causes of sterility after a first pregnancy into ten groups, based on a careful study of the 90 cases. The groups were thus arranged in order of frequency: 1. Sequelæ of inflammatory processes arising during the puerperium; 2. Catarrhal endometritis; 3. Sequelæ of inflammatory processes not puerperal in origin (43 cases came under one or other of these groups); 4. Displacements of the uterus; 5. Neoplasms of the uterus; 6. Constitutional sources of sterility established after the first pregnancy; 7. Impaired potency of the husband, well authenticated by the clinical history; this condition has more than a negative influence on the uterus and ovaries; 8. Super-involution or atrophy of the uterus; 9. New growths in the ovary; 10. Uncertain or unknown causes. Under group 6 were included cases where anæmia, cachexia, obesity, or emaciation arose after the first pregnancy. As the case could not readily be kept under observation after their discharge from the hospital, or after dispensing with the physician's services, Dr. Kleinwachter could come to no conclusions as to treatment—*Brit. Medical Journal.*

**Photophoxylin.**—Geo. M. Beringer, having attempted the manufacture of photoxylin, a substance used in Russia in place of gun-cotton, has described the method employed by him. Wood-pulp was obtained of the manufacturer both in the loose, fibrous form, and as condensed by rolling into thick and porous sheets. After careful drying, the pulp was nitrated in the following solution:

| | |
|---|---|
| Nitrous acid, 43° Baume | 2½ ℔. av. |
| Sulphuric acid | 4¼ ℔. av. |
| Potassium nitrate, granular | 8 oz. av. |

The acids having been mixed in an earthenware crock and allowed to cool to 90° F., the potassium nitrate was added and thoroughly incorporated. Four oz. av. of the well-dried pulp was immediately placed in the mixture and allowed to remain for twelve hours. It was then removed and thoroughly washed. The addition of a few drops of ammonia to the wash water facilitated the removal of the acid. Nitrocelluose thus prepared leaves little or no residue on burning and is entirely soluble in a mixture of fifty per cent. of concentrated ether and fifty per cent. alcohol. Three per cent. of this photoxylin is sufficient to make a very thick collodion which leaves a very tough film when applied. An addition of 5 drops of castor-oil to the fluidounce renders it flexible. This solution (also called "photoxylin") has the advantage over ordinary collodion of giving a stronger film.—*American Journal Pharmacy.*

**Opium Habit and Menstruation.**—Dr. E. B. Stephens writes (*Obstetric Gazette*): I find that, uniformly, women who have been for some time—as say one to two or three years—in the morphine habit, have an *entire arrest of menstruation.* Being placed under "treatment" with a successful result, very speedily the function is re-established, and continues to proceed in the normal manner.

**Antiseptic Treatment of Typhoid.**—Legroux (*Le Bull. Med.*, June 17, p. 805) has used the following treatment in a large series of cases and believes in it. To all cases a good dose of calomel is first given, then if diarrhœa is prominent—

Naphthol..................................
Bismuth..................................aa gr. xl.

Make ten powders and give one every hour in a capsule suspended in milk.

If less diarrhœa, naphthol alone in same dose.

If tendency to constipation—

Naphthol..................................
Magnes. salicylat ..........................

Ten powders as before.

Legroux finds in this treatment numerous advantages, both local and general, as, *e. g.*, disinfection of stools, diminution of meteorism and believes it affects favorably the course of the disease.—*Amer. Jour. Med. Sciences.*

**The Prevention of Ophthalmia Neonatorum.**—Ahlfeld, in the clinic at Marburg, has not had a case of pronounced *ophthalmia neonatorum* for three and one-half years, and no suppurating conjunctivitis for one and one-quarter years. This immunity he ascribes to the use of an antiseptic douche before labor; cleansing the child's eyelids as soon as the head is born; keeping the child's face as much as possible from the fluids in the vagina; and precautions in bathing the child. The face and head are never bathed in the water which cleanses the body; the eyes are bathed with clean water, by means of cotton.

Ahlfeld has no explanation for the fact that children seldom acquire the disease after the first week of life.—*Zeitschrift fur Geburtshulfe*, Band 14, Heft 2.

**The Treatment of Hydrocele.**—After one has had a large number of successes in the management of any particular disease, especially if these occur without a break, he is led to regard the method of treatment employed as one of peculiar value. This is what has induced Dr. C. H. Bedford to publish in the May number of the *Edinburgh Medical Journal* his manner of treatment of hydrocele.

The treatment which he has found almost universally applicable and successful is the tapping and thorough evacuation of the sac, injection of 2 drachms each of tincture of iodine and water, thorough shaking up of this injection so that all parts of the secreting surface may be brought in contact with the fluid, and the subsequent careful and efficient strapping of the testicle of the affected side, or, if the affection is bilateral, of each testicle separately. The puncture should be closed by cotton-wool, fixed by flexible collodion.

When there coexists a chronic orchitis or epididymitis, no injection should be made, but the tapping should be followed at once by thorough strapping. The latter should be renewed every fourth day, and continued for three weeks or a month. The main point, after the evacuation of the fluid, is the strapping of the testicle. This furnishes support to the part, affording great comfort to the patient, and prevents in great measure the reactionary swelling after an irritating injection has been used.—*The Medical Record.*

**Glycerin Suppositories for Habitual Constipation.**—Boas, in the *Deutsche med. Wochenschr.*, of June 7, 1888, states that in a large number of cases he has had good results from the use of glycerin enemata as a purgative; but in some cases, particularly those with hæmorrhoids, or in individuals with an irritable rectal mucous membrane, which readily bleeds, the use of the syringe is no slight objection, so that the injections must be intermitted or entirely refrained from. The use of the syringe is also inconvenient. For these reasons he has had prepared suppositories consisting of capsules containing 16 minims of pure glycerin, which he has used in twenty cases, with the best results. The suppositories have been found to retain their form and efficacy for many weeks. Fifteen to twenty minutes after using one there is a desire to go to stool, but without tenesmus or other discomfort, soon followed, as a rule, by a copious evacuation. The employment of glycerin per rectum seems specially indicated when, with the constipation, there exists gastric disorder.—*Medical News.*

**Yellow Fever.**—Dr. Sternberg reports good results in Havana in the treatment of yellow fever from the following: It is well known that in yellow fever urine and the vomited matters are highly acid. He also found the intestinal contents to have usually a more or less decided acid reaction. A microbe, therefore, capable of multiplying in the stomach and intestine in this disease must be able to grow in an acid medium. But aside from this theoretical reason for prescribing alkalies, the highly acid condition of the secretions furnishes an indication for such a treatment, and Dr. Sternberg has long desired an opportunity to see a thorough trial of a decidedly alkaline treatment. He therefore proposed the following formula:

℞. Sodii bicarb....................grammes x (gr. 150),
Hydrarg. chlorid. corrosiv., centigrammes ii (3-10 gr.),
Aquæ puræ........................litre i (1 quart),
M. Sig.—Fifty grammes (about 1¾ oz.) every hour; to be given *ice cold.*

The house physician at the Garcini Hospital has sent Dr. Sternberg a record of twelve cases treated by the alkaline and bichloride method, and it appears that all of them recovered. While these twelve cases were being treated, and a little before, eight cases were treated in the same institution by their methods, and five of the eight died.

The same record states that the gastric sediments appear later, and in no case have they been entirely dark. In some cases, gastric sediments (black vomit) did not appear, nor even nausea. The pains in the stomach were not so severe.

**Rules for Bathers.**—The Royal Humane Society recommends to the notice of the English public a code of rules published by the Society, and entitled "Caution to Bathers." They are as follows:

Avoid bathing within two hours after a meal.

Avoid bathing when exhausted by fatigue or from any other cause.

Avoid bathing when the body is cooling after perspiration.

Avoid bathing altogether in the open air if after having been a short time in the water, it causes a sense of chilliness with numbness of the hands and feet.

Bathe when the body is warm, provided no time is lost in getting into the water.

Avoid chilling the body by sitting or standing undressed on the banks or in boats after having been in the water.

Avoid remaining too long in the water; leave the water immediately if there is the slightest feeling of chilliness.

The vigorous and strong may bathe early in the morning on an empty stomach.

The young, and those who are weak had better bathe two or three hours after a meal—the best time for such is from two to three hours after breakfast.

Those who are subject to attacks of giddiness or faintness, and those who suffer from palpitation and other sense of discomfort at the heart, should not bathe without first consulting their medical adviser.—*Southern California Practioner.*

**Oxycyanide of Mercury the best of Antiseptics**.—Compared with corrosive chloride (*Comptes rend. d. Soc. d. Biol.*, July 6, 1888, p. 585):

1. Its solution has a slightly alkaline reaction and precipitates albumin only slightly.
2. It is less irritant than solutions of sublimate.
3. There is less absorption by tissues than in case of sublimate.
4. Solution 1-1500th does not attack, except slightly, the materials used in surgical instruments.
5. Tested by its power of keeping soup, the antiseptic power showed itself six times greater than that of the bichloride.
6. Tested by the power to destroy the micrococcus pyogenes aureus, the advantage was slightly in favor of bichloride, 1-1400th to 1-1300th.
7. Employed on suppurating surfaces or to render a mucous surface antiseptic, it furnishes much better results because of the tolerance by tissues and of feeble absorption.

The cyanide of mercury has about the same properties, but the oxycyanide is more powerful against the micrococcus pyogenes aureus.—*Amer. Jour. Med. Sciences.*

**Acetanilide—Antifebrin.**—These to substances are identical, but Squibb (*Ephemeris*, vol. iii. No. 3) observes that the name antifebrin is controlled by a patent, and consequently the price is enhanced. Acetanilide costs only half as much as antifebrin and one-eighth as much as antipyrin.—*Amer. Jour. Med. Sciences.*

